# Identification of molecular classification and gene signature for predicting prognosis and immunotherapy response in HNSCC using cell differentiation trajectories

**DOI:** 10.1038/s41598-022-24533-7

**Published:** 2022-11-27

**Authors:** Ji Yin, Sihan Zheng, Xinling He, Yanlin Huang, Lanxin Hu, Fengfeng Qin, Lunkun Zhong, Sen Li, Wenjian Hu, Jiali Zhu

**Affiliations:** The Affiliated Traditional Chinese Medicine Hospital of Southwest Medical University, Luzhou, Sichuan China

**Keywords:** Cancer, Computational biology and bioinformatics, Immunology, Oncology

## Abstract

Head and neck squamous cell carcinoma (HNSCC) is a highly heterogeneous malignancy with poor prognosis. This article aims to explore the clinical significance of cell differentiation trajectory in HNSCC, identify different molecular subtypes by consensus clustering analysis, and develop a prognostic risk model on the basis of differentiation-related genes (DRGs) for predicting the prognosis of HNSCC patients. Firstly, cell trajectory analysis was performed on single-cell RNA sequencing (scRNA-seq) data, four molecular subtypes were identified from bulk RNA-seq data, and the molecular subtypes were predictive of patient survival, clinical features, immune infiltration status, and expression of immune checkpoint genes (ICGs)s. Secondly, we developed a 10-DRG signature for predicting the prognosis of HNSCC patients by using weighted correlation network analysis (WGCNA), differential expression analysis, univariate Cox regression analysis, and multivariate Cox regression analysis. Then, a nomogram integrating the risk assessment model and clinical features can successfully predict prognosis with favorable predictive performance and superior accuracy. We projected the response to immunotherapy and the sensitivity of commonly used antitumor drugs between the different groups. Finally, we used the quantitative Reverse Transcription-Polymerase Chain Reaction (qRT-PCR) analysis and western blot to verify the signature. In conclusion, we identified distinct molecular subtypes by cell differentiation trajectory and constructed a novel signature based on differentially expressed prognostic DRGs, which could predict the prognosis and response to immunotherapy for patients and may provide valuable clinical applications in the treatment of HNSCC.

## Introduction

Head and neck squamous cell carcinoma (HNSCC) ranks as the 6th most common malignancy in the world, causing around 350,000 deaths annually, with major risk factors including alcohol consumption, smoking, and human papilloma virus infection^1,2^. While conventional therapeutic strategies for HNSCC, consisting of surgery, radiotherapy, chemotherapy, targeted therapy, and immunotherapy, have advanced significantly, the 5-year survival rate has barely increased and remained below 50%^3^. Some of the patients with similar tumor, node, and metastasis (TNM) stages have different clinical characteristics and outcomes, which may be due to the heterogeneity of HNSCC^4^. Therefore, an in-depth exploration at the molecular level is urgently needed for accurate therapeutic and prognostic assessments.

Intra-tumoral heterogeneity is characterized by novel genetic mutations and molecular phenotypes that emerge in each filial generation during the malignant transformation of the primary cells, and thus tumors are regarded as a collection of various tumor cell clones^5,6^. The reason for the different clinical features and outcomes of HNSCC patients with similar TNM stages can be attributed to intra-tumor heterogeneity. Therefore, it is crucial to elucidate the intra-tumor heterogeneity of HNSCC. Traditional bulk RNA-sequencing (RNA-seq), which analyzes the total transcriptome of the tissue sample, offers information about the average expression level of each gene in a mixture of cell populations, but it does not take into account the cellular heterogeneity in the sample^7,8^. With the advancement of cell isolation and sequencing technologies, single-cell RNA-seq (scRNA-seq) has offered an efficient way to unravel the characteristics of the single-cell transcriptome, which opens up novel paths to study intra-tumor heterogeneity^9^.

Puram et al. analyzed the ecosystem of head and neck cancers by Smart-Seq technology, depicting the first cellular map of head and neck cancers and revealing the types of head and neck tumors and their metastasis-related programs^10^. In the study, we determined four molecular subtypes of HNSCC by cell differentiation trajectory based on scRNA-seq, and the subtypes are predictive of clinical prognosis, immune infiltration status, and immunotherapeutic response in HNSCC patients. Then, we developed a differentiation-related genes (DRGs) signature and a nomogram integrating the signature and prognostic clinical features for accurate prognosis prediction of HNSCC patients. Conclusively, we provided a novel perspective on the advancement of therapeutic approaches and the assessment of prognosis for HNSCC by investigating the cell differentiation trajectory and exploring its relationship with clinical outcomes and response to immunotherapy.

## Result

### Quality control and normalization

**Figure 1:**
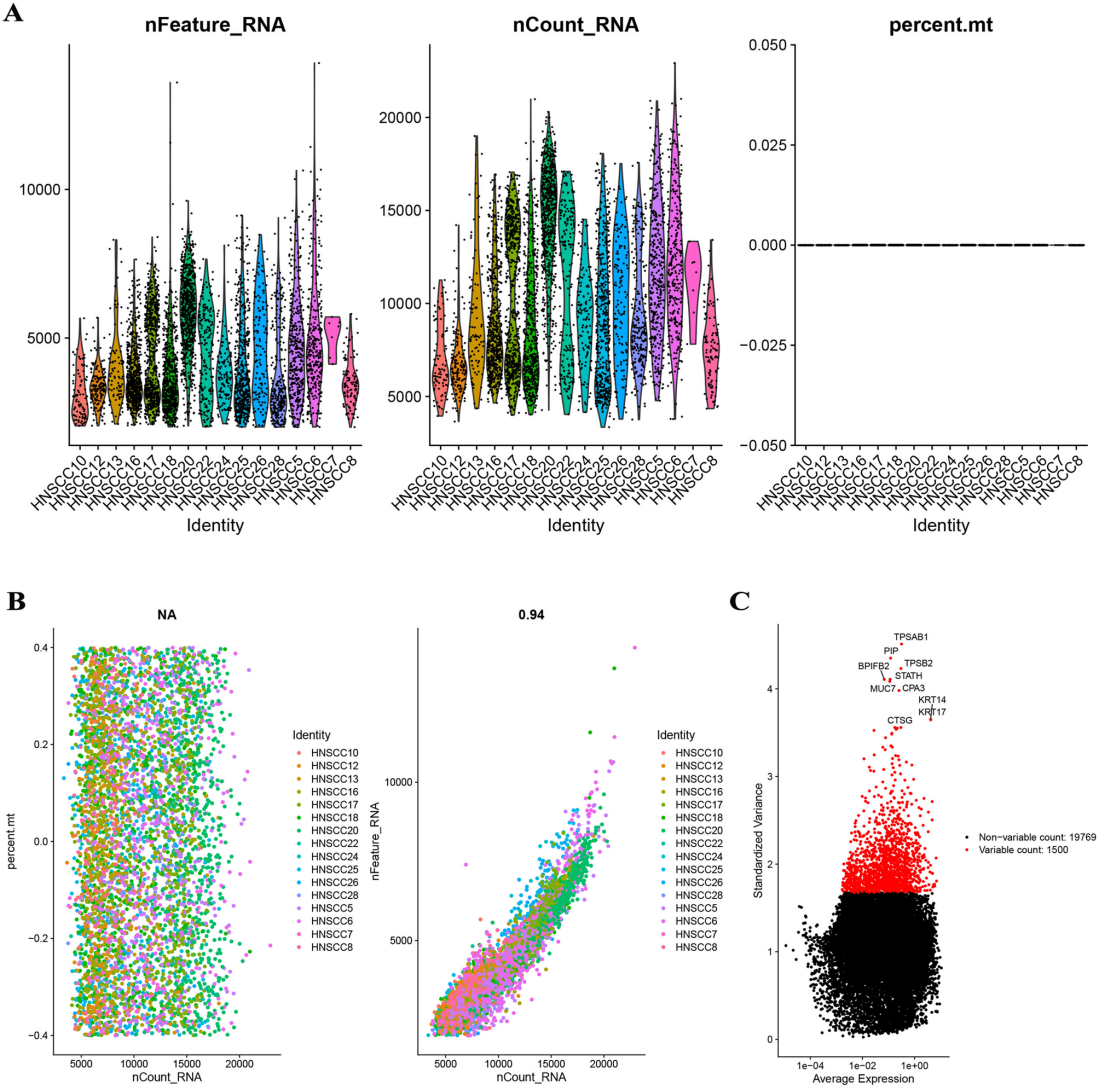
Quality control and normalization of scRNA-seq data from GSE103322. (**A**) After quality control and normalization, 4873 cells were extracted for further study. (**B**) Sequencing depth had no link with mitochondrial gene sequences and had a substantial positive correlation with total intracellular sequences. (**C**) There were 19,785 genes studied, with 1500 having high intercellular variation and 18,285 having low variation.

In this study, 4873 cells were extracted from GSE103322 for further study after quality control and standardization (Fig. 1A). Sequencing depth had no link with mitochondrial gene sequences and had a substantial positive correlation with total intracellular sequences (R = 0.94, Fig. 1B). There were 19,785 genes studied, with 1500 having high intercellular variation and 18,285 having low variation (Fig. 1C).

### Dimensionality reduction and cell trajectory analysis

**Figure 2:**
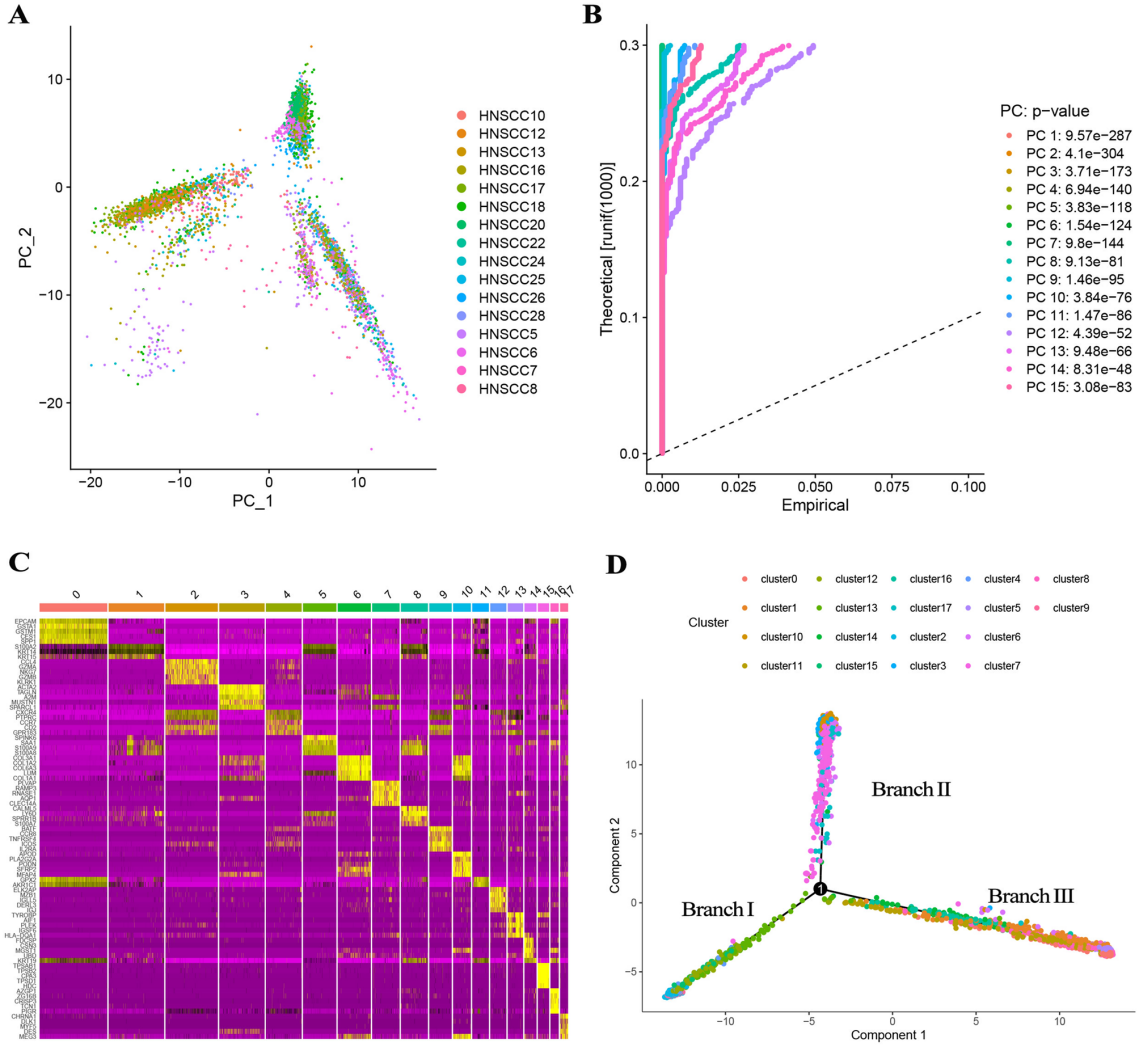
Dimensionality reduction and cell trajectory analysis based on scRNA-seq data from GSE103322. (**A**) PCA on the basis of scRNA-seq data. (**B**) p < 0.5 was used to identify 15 PCs with significant differences. (**C**) 4873 HNSCC cells were divided into 18 clusters, a total of 4363 marker genes were recognized using differential analysis, and the top 5% of marker genes in each cluster were visualized on the heat map by using the tSNE algorithm. (**D**) Pseudotime and trajectory analysis.

We performed a preliminary dimensionality reduction of scRNA-seq data using PCA analysis, which revealed no substantial segregation among HNSCC cells, hence the 15 PCs with remarkable differences were chosen for further investigation (Fig. 2A,B). Then, 4873 HNSCC cells were divided into 18 clusters, a total of 4363 marker genes were recognized using differential expression analysis, and the top 5% of marker genes in each cluster were visualized on the heat map by using the tSNE algorithm (Fig. 2C). According to pseudotime and trajectory analyses, clusters 2/4/14/16 were primarily dispersed in subset I, clusters 3/7/8/17 were primarily dispersed in subset II, clusters 0/1/9/15 were primarily dispersed in subset III, and clusters 5/6/17 were primarily dispersed in the subsets II/III, and clusters 10/13 were dispersed in the three subsets (Fig. 2D).

### Molecular functional analysis

Subset I included 552 DRGs, subset II had 375 DRGs, and subset III had 399 DRGs, for a total of 818 DRGs (Supplementary Table S1). According to GO and KEGG enrichment analyses, the DRGs in subsets I were engaged in differentiation, cell–cell adhesion, and immunological responses (Supplementary Figure S1A and S1B; Supplementary Table S2), the DRGs in subsets II were engaged in immune killing and cell–cell adhesion (Supplementary Figure S1C,D; Supplementary Table S3), and the DRGs in subsets III were involved in inflammation, differentiation, immunological responses, and cell–cell adhesion (Supplementary Figure S1E,F; Supplementary Table S4).

### Construction of molecular subtypes

**Figure 3:**
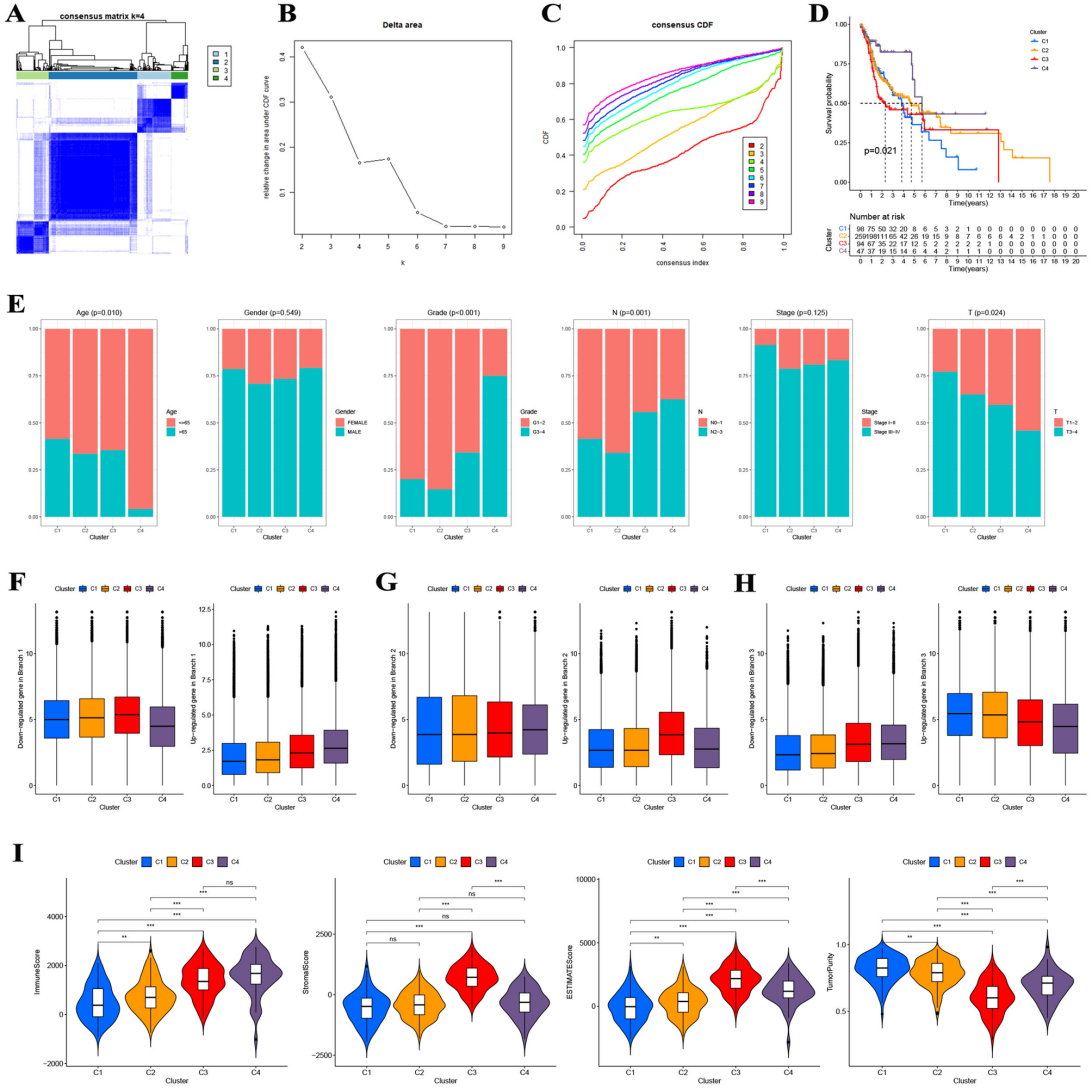
Consensus clustering analysis of HNSCC patients from the TCGA. (**A–C**) Four molecular subtypes were recognized at a clustering threshold of K = 9. (**D**) The Kaplan–Meier analysis indicated the statistically significant results of consensus clustering for HNSCC. (**E**) The four molecular subtypes were statistically related to age, pathological stage, N stage, and T stage of HNSCC patients, but the differences with gender distribution and clinical stage were not statistically significant. (**F–H**) The down-or up-regulated DRGs in subsets I/II/III had a similar expression pattern in clusters 4/3/1 and 2. (**I**) Different tumor microenvironment scores and tumor purity in the four molecular subtypes.

A DRG-based consensus clustering analysis was conducted, and at a clustering threshold of maxK = 9, four molecular subtypes containing all the HNSCC samples were identified (Fig. 3A–C and Supplementary Figure S2). The Kaplan–Meier analysis indicated the statistically significant results of consensus clustering for HNSCC, with cluster 4 having the highest survival rate and cluster 1 having the lowest (Fig. 3D, p = 0.021). The four molecular subtypes were significantly related to age (p = 0.010), pathological stage (p < 0.001), N stage (p = 0.001), and T stage (p = 0.024) of HNSCC patients, but no statistical differences were found in the distribution of gender and clinical stage among the four subtypes (Fig. 3E). Furthermore, the down-or up-regulated DRGs in subsets I/II/III had a similar expression pattern in clusters 4/3/1 and 2, indicating that clusters 4/3/1 and 2 were made up of subsets I/II/III independently (Fig. 3F–H).

### Evaluation of immunological characteristics

**Figure 4:**
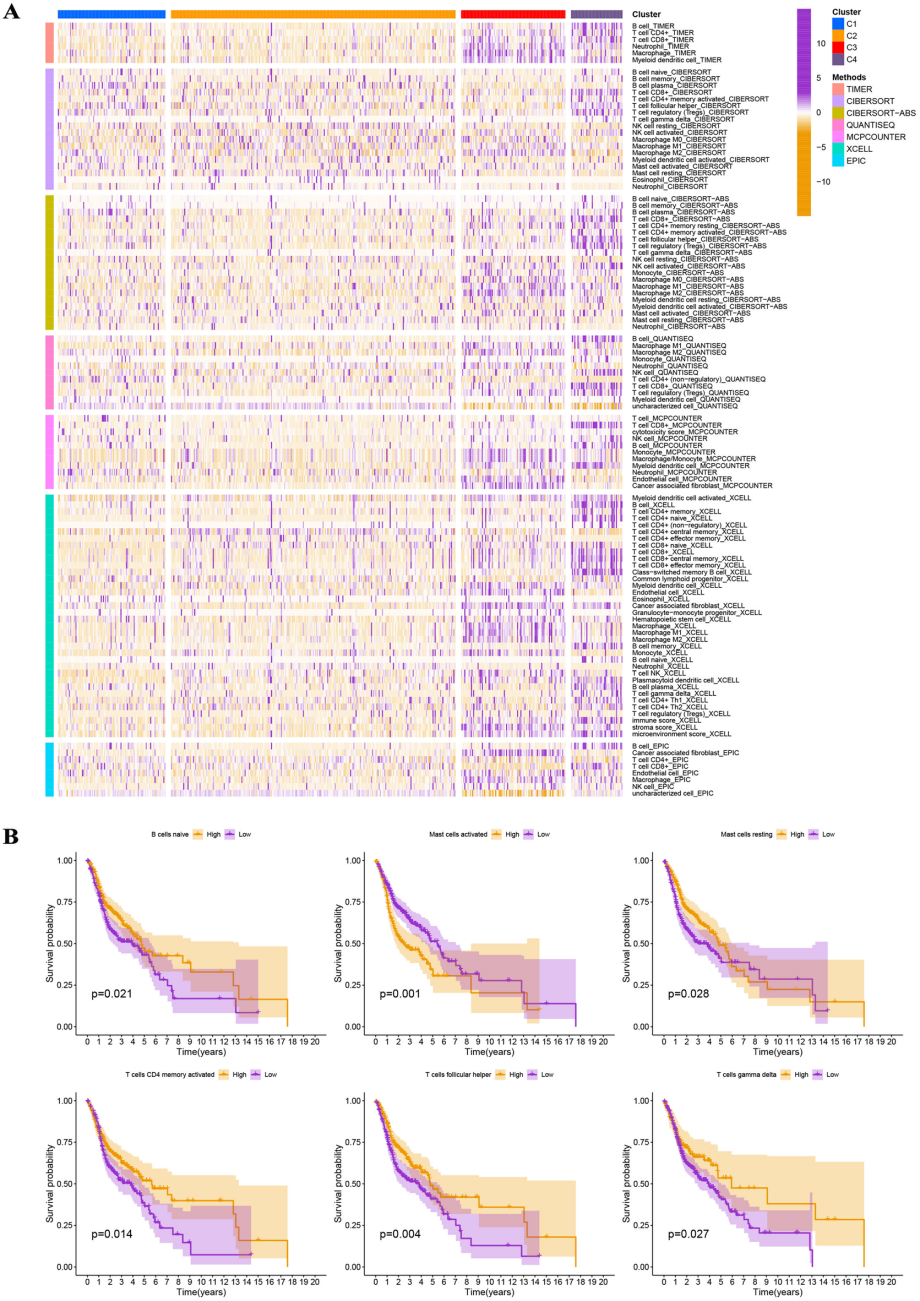
Comprehensive evaluation of immunological characteristics. (**A**) There were statistically significant differences in the content of most of the immune cells in the four clusters. (**B**) Kaplan–Meier analysis indicated that some immune cells were also statistically related to survival, including B cells naive, mast cells activated, mast cells resting, plasma cells, T cells CD4 memory activated, T cells follicular helper, and T cells gamma delta.

**Figure 5:**
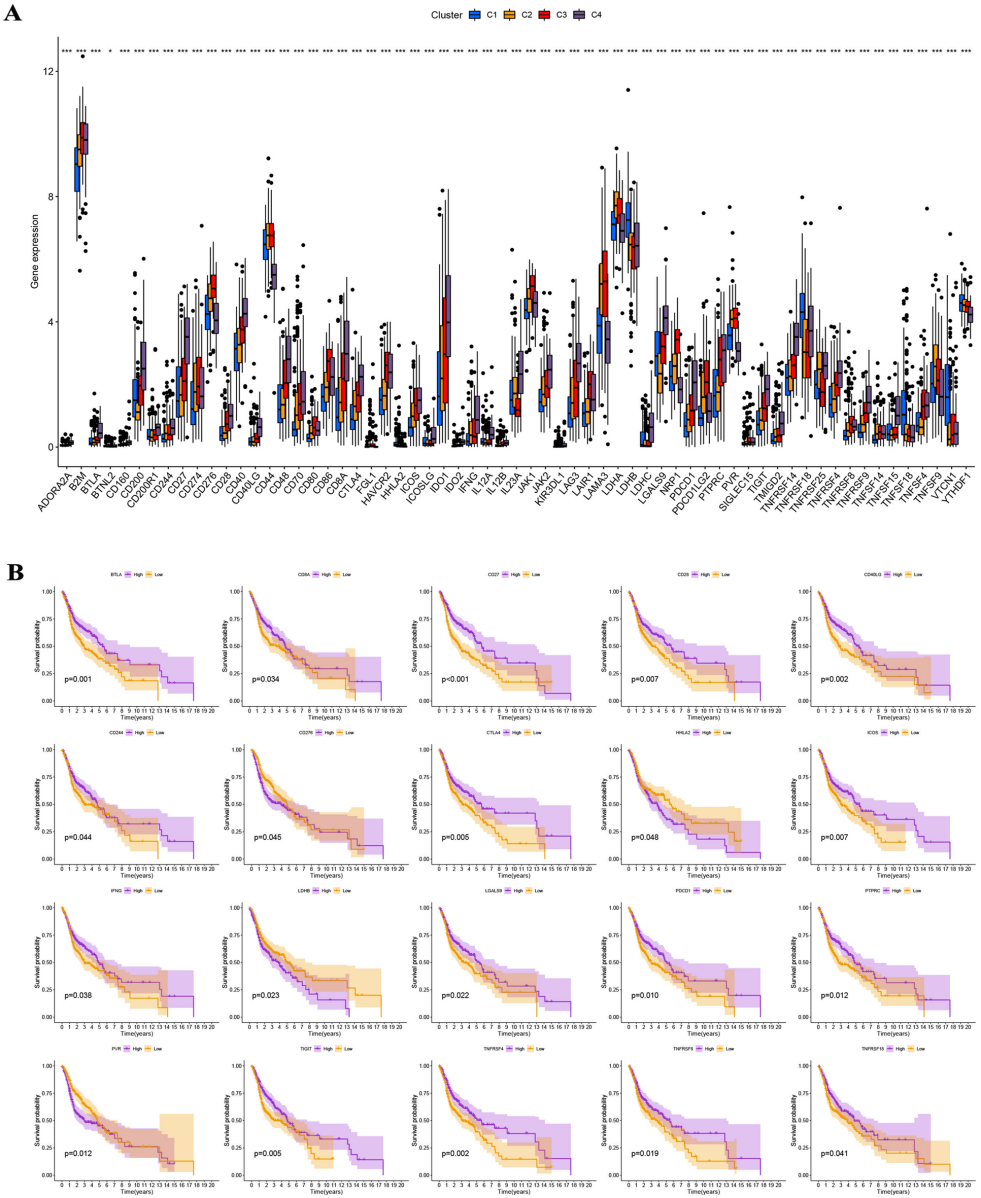
Expression levels of ICGs between four molecular subtypes and survival analysis. (**A**) The expression of some ICGs, including BTLA, CD274, CTLA-4, LAG3, PDCD1, and TIGIT, among others, was significantly different between the four clusters. (**B**) Kaplan–Meier analysis indicated that some ICGs were also significantly associated with survival, including BTLA, CTLA-4, PDCD1, and TIGIT.

On the basis of tumor microenvironment scores, we revealed that immune scores increased in turn in clusters 1/2/3/4, and cluster 4 has the highest stromal scores (Fig. 3I). Moreover, estimates scores in cluster 3/4/2/1 steadily declined, whereas tumor purity increased in turn in cluster 3/4/2/1 (Fig. 3I). On the basis of XCELL, TIMER, QUANTISEQ, MCPCOUNTER, EPIC, CIBERSORT-ABS, and CIBERSORT, we discovered statistical differences in the content of most of the immune cells in the four clusters (Fig. 4A). Kaplan–Meier analysis indicated that some immune cells were also significantly related to survival, including B cells naive, mast cells activated, mast cells resting, plasma cells, T cells CD4 memory activated, T cells follicular helper, and T cells gamma delta (Fig. 4B). We also found that the expression of some ICGs, including BTLA (p < 0.001), CD274 (p < 0.001), CTLA-4 (p < 0.001), LAG3 (p < 0.01), PDCD1 (p < 0.001), and TIGIT (p < 0.001), among others, was also significantly different between the four clusters (Fig. 5A). Kaplan–Meier analysis indicated that ICGs were also significantly associated with survival, including BTLA (p = 0.001), CTLA-4 (p = 0.005), PDCD1 (p = 0.010), and TIGIT (p = 0.005) (Fig. 5B).

### Establishment of risk model

**Figure 6:**
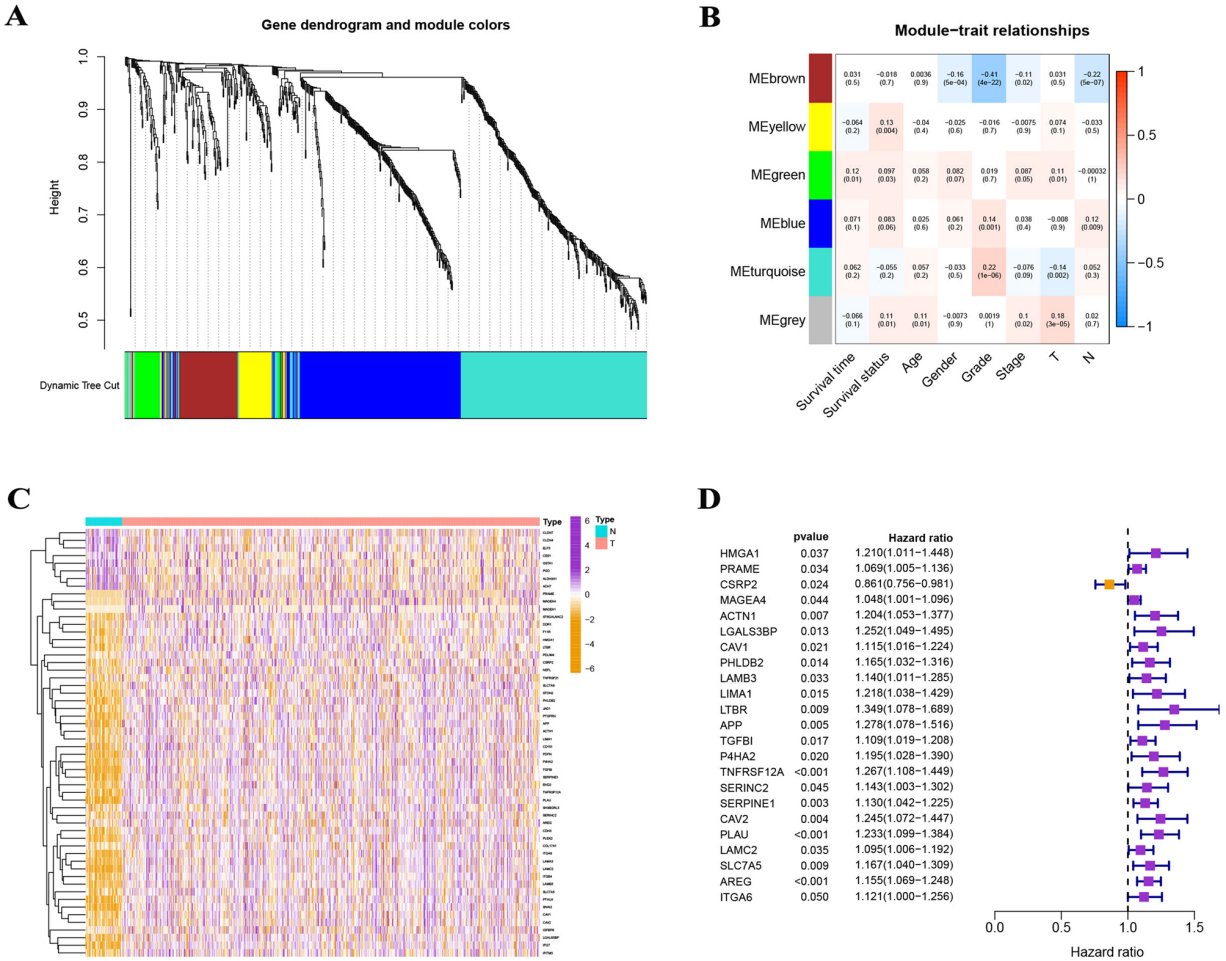
Establishment of a risk model by using WGCNA, differential expression analysis, univariate Cox analysis, and multivariate Cox analysis. (**A,B**) With a soft threshold of = 4, six modules were accessible, three of which (green, grey, and yellow modules) were strongly associated with survival status. The numbers above the parentheses indicate the correlation coefficient, and the numbers in parentheses represent the p-value. (**C**) A total of 56 differentially expressed DRGs were acquired from the 3 modules. (**D**) 23 prognostic DRGs were further identified by univariate analysis.

749 DRGs were used for WGCNA following the intersection of DRGs in the TCGA and GSE117973 sets. With a soft threshold of = 4, six modules were accessible, three of which (green, grey, and yellow modules) were strongly associated with survival status (Fig. 6A,B). 56 differentially expressed DRGs were acquired from the 3 modules, and 23 prognostic DRGs were further identified by univariate Cox analysis and included in multivariate Cox analysis (Fig. 6C,D). Finally, a 10-DRG prognostic signature was constructed, and risk scores for each sample were generated using the relative coefficient and gene expression. Based on the median risk score, 249 samples were categorized into the high-risk group and the remaining 249 samples into the low-risk group in the TCGA set; 34 samples were categorized into the high-risk group and the remaining 43 samples into the low-risk group in the GSE117973 set.

**Figure 7:**
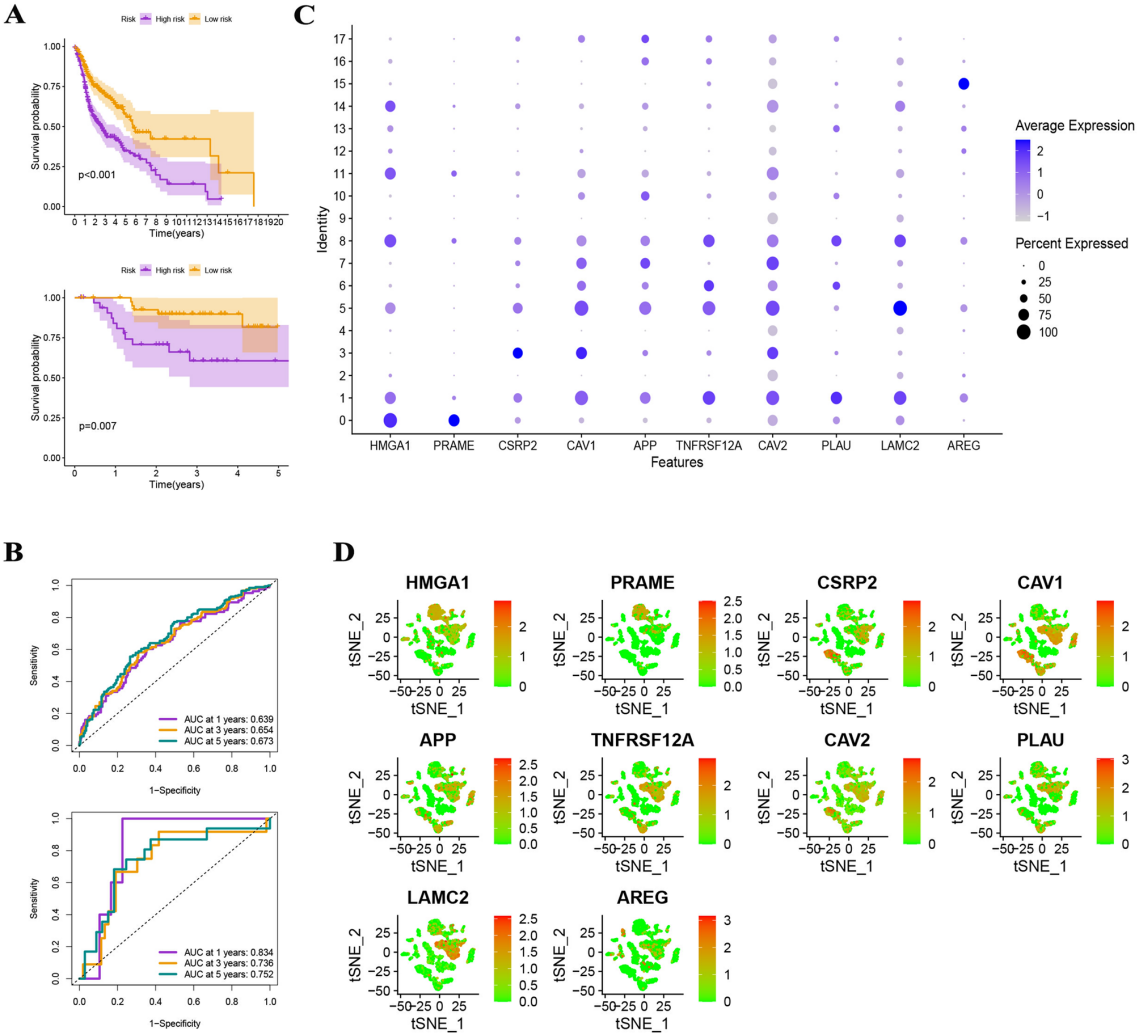
Validation of the risk assessment model. (**A**) Survival rates in high-risk HNSCC patients (TCGA and GEO) were considerably lower than in low-risk individuals. (**B**) In the TCGA collection, the ROC curves for predicting 1-year, 3-year, and 5-year AUC values were 0.639, 0.654, and 0.673, respectively, and in the GSE117973 set, they were 0.834, 0.736, and 0.752, respectively. (**C**) The expression levels of 10 DRGs.

**Figure 8:**
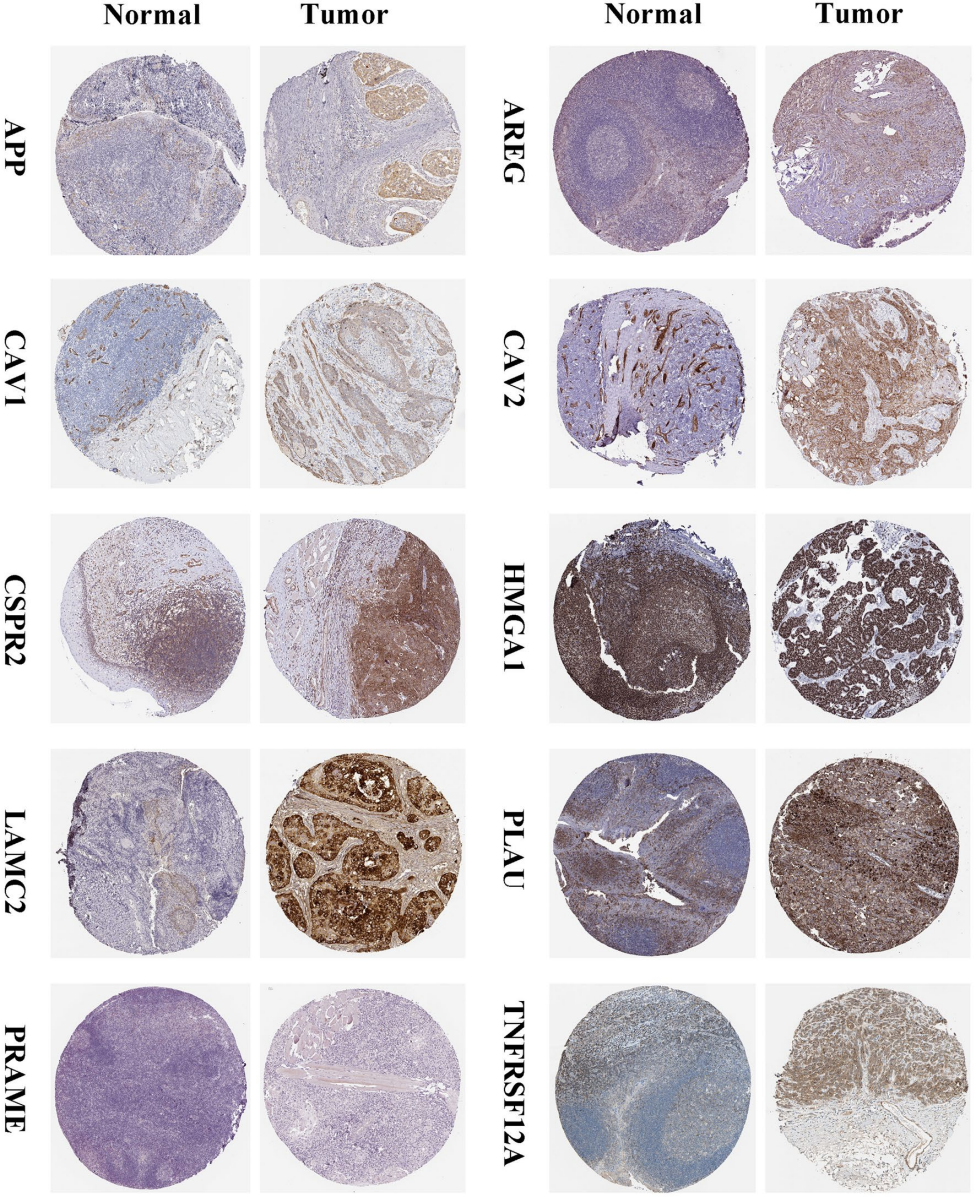
On the basis of the HPA database, the protein expression levels of CSRP2, CAV1, APP, TNFRSF12A, CAV2, PLAU, and LAMC2 were significantly higher in HNSCC tumor tissues than in normal tissues, whereas the protein expression levels of HMGA1, PRAME, and AREG were not significantly different between normal and tumor tissues.

According to a Kaplan–Meier study, survival rates in high-risk HNSCC patients (TCGA and GEO) were considerably lower than in low-risk individuals (Fig. 7A). In the TCGA collection, the ROC curves for predicting 1-year, 3-year, and 5-year AUC values were 0.639, 0.654, and 0.673, respectively, and in the GSE117973 set, they were 0.834, 0.736, and 0.752, respectively (Fig. 7B). In addition, the expression levels of 10 DRGs are depicted in Fig. 7C,D. On the basis of the HPA database, the protein expression levels of CSRP2, CAV1, APP, TNFRSF12A, CAV2, PLAU, and LAMC2 were significantly higher in HNSCC tumor tissues than in normal tissues, whereas the protein expression levels of HMGA1, PRAME, and AREG were not significantly different between normal and tumor tissues (Fig. 8).

### Configuration of nomogram

**Figure 9:**
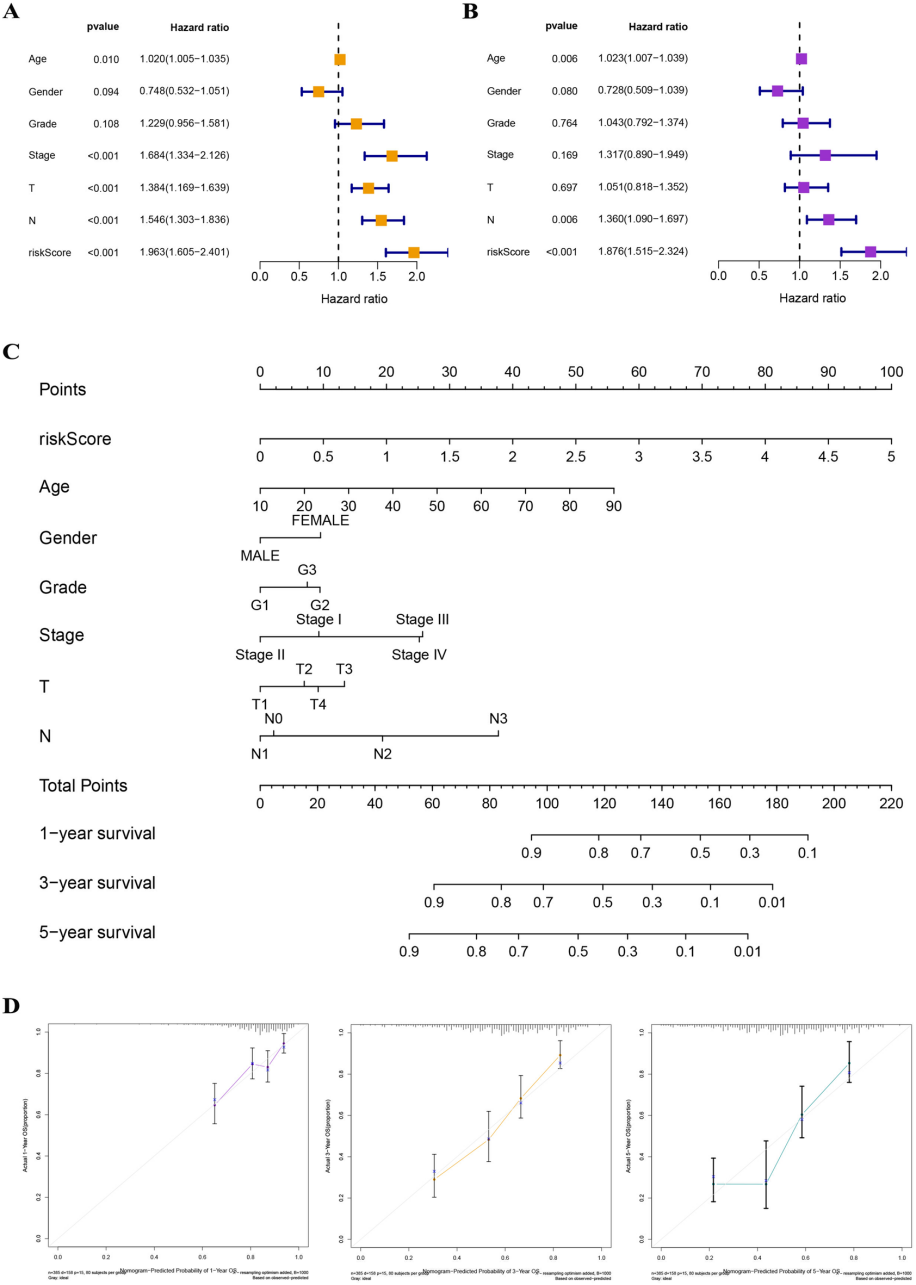
Construction of a nomogram. (**A,B**) Univariate Cox analysis demonstrated that clinical factors, including risk score, age, clinical stage, T stage, and N stage, were statistically correlated to prognosis, and the multivariate Cox analysis demonstrated that risk score, age, and N stage were independent prognostic risk factors. (**C**) A nomogram for predicting 1-year, 3-year, and 5-year prognosis. (**D**) The calibration curves for predicting 1-year, 3-year and 5-year prognosis.

Univariate Cox analysis demonstrated that clinical features, including risk score [HR 1.963, 95% CI (1.6.05–2.401), p value < 0.001], age [HR 1.020, 95% CI (1.005–1.035), p value = 0.010], clinical stage [HR 1.684, 95% CI (1.334–2.126), p value < 0.001], T stage [HR 1.384, 95% CI (1.169–1.639), p value < 0.001], and N stage [HR 1.546, 95% CI (1.303–1.836), p value < 0.001] were remarkably associated with prognosis, and the corresponding multivariate Cox analysis demonstrated that risk score [HR 1.876, 95% CI (1.515–2.324), p value < 0.001], age [HR 1.023, 95% CI (1.007–1.039), p value = 0.006], and N stage [HR 1.360, 95% CI (1.090–1.697), p value = 0.006] also were independent prognostic risk factors (Fig. 9A,B). The 10-DRG prognostic signature was integrated with clinicopathologic characteristics to develop a nomogram for predicting prognosis, and the calibration curves for predicting 1-year, 3-year, and 5-year prognosis were in favorable accordance with the actual values (Fig. 9C,D).

### Evaluation of immunological characteristics and therapeutics

**Figure 10:**
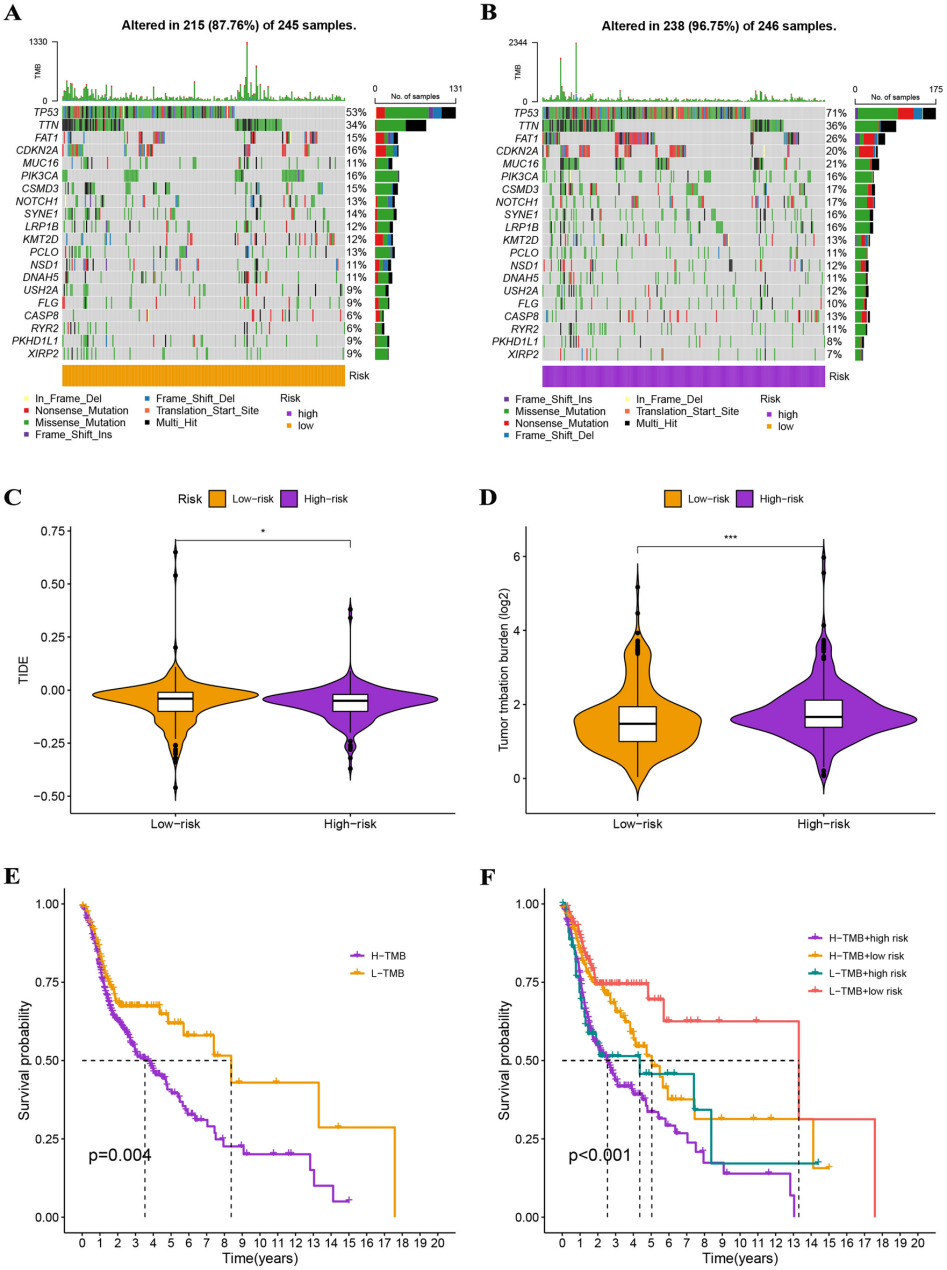
Prediction of immunotherapy response. (**A,B**) Gene mutation analysis indicated that more genes were mutated in the high-risk group. (**C,D**) TIDE scores were lower in the high-risk group, and TMB scores were higher in the high-risk group. (**E**) Survival rates were significantly higher in the L-TMB groups. (**F**) Survival rates were significantly different between the four groups.

Gene mutation analysis indicated that the mutation rate in the high-risk group (96.75%) was significantly higher than that in the low-risk group (87.76%), and the top 20 genes with the highest mutation frequency are presented in Fig. 10A,B. TIDE scores were lower in the high-risk group than in the low-risk group (p < 0.05), and TMB scores were higher in the high-risk group than in the low-risk group (p < 0.001), suggesting that patients in the high-risk group may be more sensitive to immunotherapy (Fig. 10C,D). The Kaplan–Meier analysis showed that the survival time was significantly longer in the L-TMB group than in the H-TMB group, the survival rate was lowest in the H-TMB + high-risk group, and the survival rate was highest in the L-TMB + low-risk group, indicating that both the TMB and the risk score accurately predicted the prognosis of HNSCC patients (Fig. 10E,F).

### Identification of novel potential compounds

Besides predicting the response of different groups to immunotherapy, we also attempted to identify common anti-tumor drugs that target our signature treatment of HNSCC patients. We discovered that the IC50 of 26 common anti-tumor drugs used in HNSCC therapy was statistically different in different groups (Supplementary Figure S3).

### Validation of risk model

**Figure 11:**
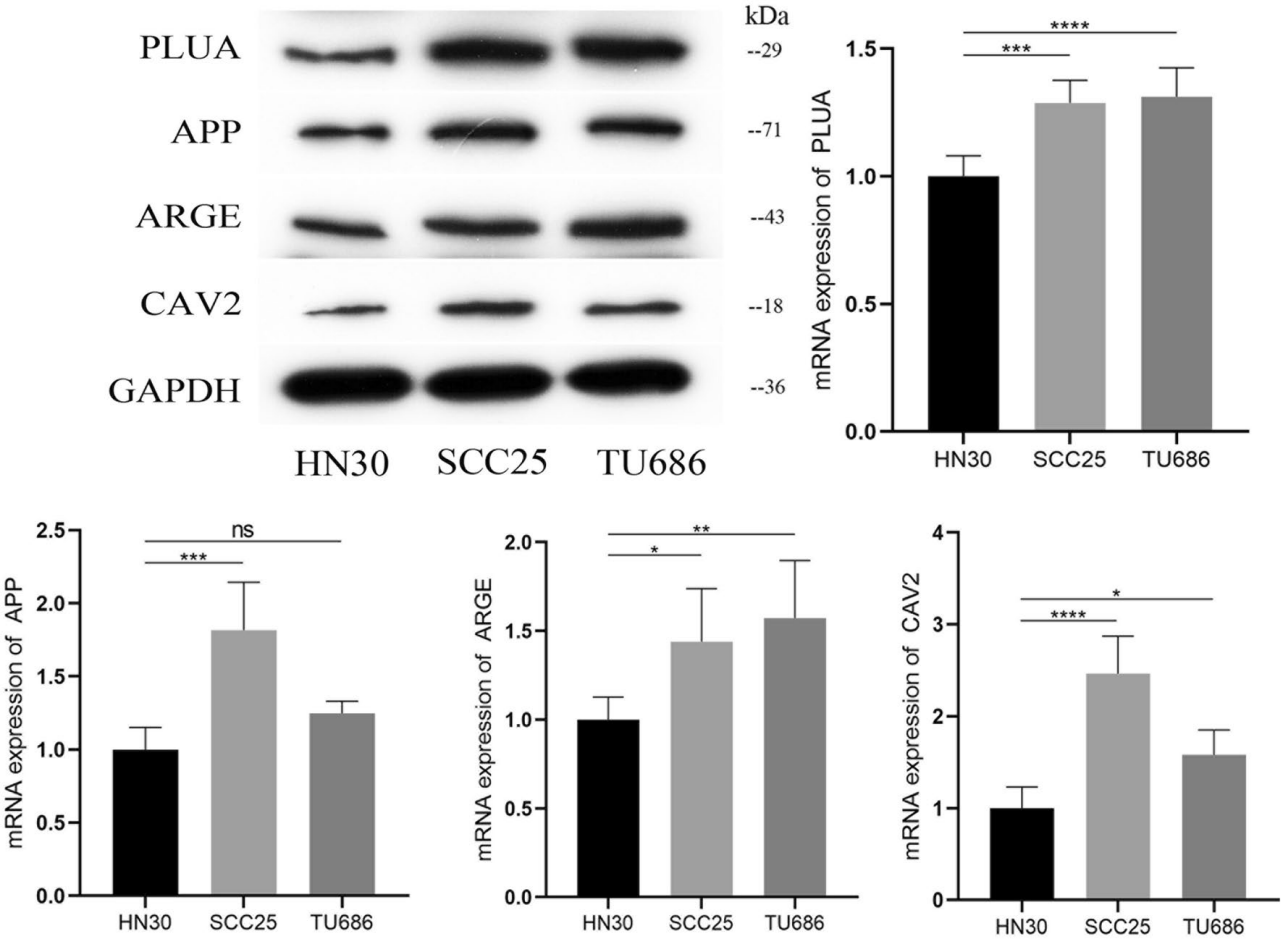
The mRNA and protein expression levels of PLAU, APP, AREG and CAV2 were upregulated in cancer cells compared to normal cells.

To further validate our results, we used the quantitative Reverse Transcription-Polymerase Chain Reaction (qRT-PCR) analysis and western blot to verify the expression levels of mRNA and protein inWSU-HN30 and TU686 and SCC25 cells of 4 DRGs (PLAU, APP, AREG and CAV2). The results exhibited that the mRNA and protein expression levels of PLAU, APP, AREG and CAV2 were upregulated in cancer cells compared to normal cells (Fig. 11 and Supplementary Figure S4). The above results confirm the reliability and stability of our analytical method.

## Discussion

Previous studies have evaluated the response to therapy and prognosis of HNSCC patients mainly based on tumor site, pathological stage, TNM stage, age, gender, living habits, and physical condition^11–15^. However, due to the heterogeneity of HNSCC, even patients with these similar characteristics mentioned above are treated in a similar modality, yet end up with very different clinical outcomes^16^. Therefore, the relationship between the heterogeneity of HNSCC and treatment response and prognosis deserves further investigation. In the study, we identified 4 molecular subtypes of HNSCC by cell differentiation trajectory, developed a signature based on differentially expressed prognostic DRGs, and constructed a nomogram integrating the signature and prognostic clinical features for predicting clinical outcomes and response to immunotherapy.

During the malignant transformation of primary cells, new genetic mutations and molecular phenotypes emerge with each generation of offspring, leading to differences in clinical characteristics, therapeutic response, drug resistance, and prognosis of patients^17–19^. In the research, HNSCC cells in different differentiation phases were divided into four subsets on the basis of cell trajectory analysis. These findings suggest that the activation or inhibition of multiple signaling pathways is linked to HNSCC cell differentiation. Finally, we established a baseline for the examination of intra-tumoral heterogeneity and the detection of distinct molecular phenotypes in HNSCC using WGCNA, GO, and KEGG enrichment analysis.

Distinct molecular phenotypes and subtypes of HNSCC can assist in disease diagnosis, instruct treatment strategies, facilitate the development of precision medicine, and boost the development of novel models, which have a crucial role in better understanding of HNSCC development and treatment^20^. Chen et al. classify HNSCC subtypes as exhausted, active, and non-immune classes, which might aid in tailoring the optimal immunotherapy strategy^21^. Sen et al. recognized 2 prognostic and clinical immune subtypes of HNSCC, which differed in various aspects of prognosis, clinical outcomes, immune features, mutation pattern, biological functions, and response to immune checkpoint blockade therapy^22^. Zhang et al. characterized 3 HNSCC subtypes with different molecular profiles and survival outcomes and proposed therapeutic drugs that might be suitable for specific subtypes^23^. Therefore, the exploration of individualized treatment modalities for HNSCC based on molecular subtypes is worthwhile. We have constructed DRG-based molecular subtyping, with cluster 4 having the highest survival rate and cluster 1 having the lowest. Numerous researches have shown that M0 macrophages are associated with poor prognosis^24,25^. On the contrary, the high density of CD8^+^ T cells, regulatory T cells, B cells, and M1 macrophages predicted a good prognosis^26–30^. These findings are consistent with our findings. In addition, the proportion of ICGs is higher in cluster 4 than in cluster 1, suggesting that cluster 4 tumors are associated with immune escape and immunosuppression. Therefore, HNSCC patients with cluster 4 may be better able to benefit from immune checkpoint inhibitors with better efficacy^31,32^.

Different molecular subtypes of HNSCC based on the DRGs exhibited different survival profiles, which indicated that DRG-based classification could be effective in predicting patient prognosis. Therefore, we developed a DRG-based prognostic signature and verified it to be highly accurate and efficient. And, this is the first DRG-based model developed by multivariate Cox analysis for predicting prognosis and response to immunotherapy in HNSCC patients. In addition, we constructed a nomogram integrating the DRG-based signature and prognostic clinical features, providing a visualization for predicting patient prognosis that is more accurate and efficient than using the signature alone. TIDE scores were lower in the high-risk group than in the low-risk group, and TMB scores were higher in the high-risk group than in the low-risk group, suggesting that patients in the high-risk group may be more sensitive to immunotherapy. Besides predicting the response of different groups to immunotherapy, we also attempted to identify common anti-tumor drugs that target our signature treatment of HNSCC patients, such as Docetaxel, Gemcitabine, Methotrexate, Vinblastine, and others.

Nonetheless, the current study has some shortcomings and limitations. First, the results may be biased as the majority of samples from TCGA are non-metastatic. Second, various clinicopathological characteristics are not better described, such as alcohol consumption and smoking, resulting in a limited number of characteristics involved in the nomogram. As a result, we plan to collect additional clinical samples, expand our sample size and closely follow up our results to further examine and validate our model.

## Conclusion

In summary, we identified distinct molecular subtypes by cell differentiation trajectory and constructed a novel signature based on differentially expressed prognostic DRGs, which could predict the prognosis and response to immunotherapy for patients and might present valuable clinical applications in the treatment direction of HNSCC.

## Methods

### Preparation of data

The scRNA-seq data of HNSCC were obtained from the GSE103322 set in the Gene Expression Omnibus (GEO, https://www.ncbi.nlm.nih.gov/geo/)^10^. Data processing and visualization were conducted with the R package “Seurat” (v3.2.1) running on R software (v4.0.3)^33^. The PercentageFeatureSet function was employed to count the percentage of mitochondrial genes for each cell^34,35^. A correlation analysis between sequencing depth and the length of mitochondrial gene sequences or gross intracellular sequences detected. Cells with an intracellular gene number < 50, intracellular sequencing number < 3, and mitochondrial gene fraction > 5% were excluded. The LogNormalize method was applied to normalize the scRNA-seq data, and the variance analysis was utilized to recognize the top 1500 genes with highly variable features. The bulk RNA-seq data and clinical information of HNSCC were extracted from the GSE117973 dataset and The Cancer Genome Atlas (TCGA, https://tcga-data.nci.nih.gov/tcga)^36,37^. By excluding duplicate and missing follow-up time data, the relevant data was retrieved.

### Dimensionality reduction

Based on the standard of a false discovery rate (FDR) < 0.05, the separated remarkable dimensions were extracted by principal component analysis (PCA), and the top 15 principal components (PCs) were dimensionalized by the t-distributed stochastic neighbor embedding (tSNE) algorithm to acquire the main clusters. In addition to the conventional dimensionality reduction methods mentioned above, many novel single-cell computational tools have emerged, such as the DomainAdversarial and Variational Auto-Encoder, the Potential of Heat-diffusion for Affinity-based Transition Embedding, etc.^38,39^. Under the condition of |log2(FC)|> 1 and FDR < 0.05, the marker genes in each cluster were obtained and the top 5 marker genes in the clusters were listed in the heat map.

### Cell trajectory analysis and molecular functional analysis

The R package “Monocle” was employed to conduct the pseudotime and cell differentiation trajectory analyses^40^. Differential expression analysis was conducted on cells with different differentiation status to identify DRGs by the R package “limma” (|log2(FC) |> 1 and FDR < 0.05)^41^. The R package “clusterProfiler” was employed for Gene Ontology (GO) and Kyoto Encyclopedia of Genes and Genomes (KEGG) analysis^42^.

### Identification of molecular subtypes

The R package “ConsensusClusterPlus” was applied for consensus unsupervised clustering analysis to divide HNSCC patients from TCGA into different molecular subtypes according to DRG expression^43^. The K-means algorithm and cumulative distribution function curve were used to identify the best number of subtypes, and 50 iterations with maxK = 9 were applied for steady subtypes. The Kaplan–Meier analysis with log-rank tests was performed to assess the difference in survival between the distinct molecular subtypes, the proportion of clinical features in each distinct molecular subtype was displayed, and the expression levels of DRGs in specific molecular subtypes were evaluated within different cell differentiation trajectories^44^.

### Exploration of immunological characteristics

For each sample in the four molecular subtypes, immune, stromal, and ESTIMATES scores were calculated for each sample. The content and infiltration density of immune cells for each sample in the four molecular subtypes were identified. Meanwhile, we studied the relationship between the four molecular subtypes and the expression level of immune checkpoint genes (ICGs). The prognostic value of immune cells and ICGs was investigated by using Kaplan–Meier analysis with log-rank tests.

### Establishment and validation of risk model

We perform model construction and validation using the TCGA cohort as the training set and the GSE117973 cohort as the validation set. These DRGs in the TCGA and GSE117973 cohorts were intersected, and the expression profiles were subjected to normalization and batch correction. By analyzing correlation analysis using weighted correlation network analysis (WGCNA), we recognized modules significantly related to the survival status of HNSCC patients. The genes in the crucial modules were progressively subjected to differential expression analysis (with | log2 (FC) |> 0.585 and FDR < 0.05), univariate Cox regression analysis, multivariate Cox regression analysis, and finally a DRGs prognostic model was developed. The concrete risk score for each patient was calculated, and the risk score formula was as follows:

$$\sum\nolimits_{i = 1}^{k} {\beta_{i} S_{i} }$$

On the basis of the median risk score, HNSCC patients were classified into high-and low-risk groups. We performed Kaplan–Meier survival analysis to evaluate the prognostic ability of the prognostic model in the TCGA and GEO cohorts. The time-dependent receiver-operating characteristic (ROC) curves and the area under the curve (AUC) were applied to assess the predictive ability of the model for survival. To further validate the model, we employed the Human Protein Atlas (HPA: https://www.proteinatlas.org/) database to investigate the protein levels of these DEGs in normal and tumor tissues.

### Configuration of nomogram

Univariate and multivariate Cox analyses were applied to confirm that the risk score was an independent predictor of clinical prognosis. A nomogram integrating prognostic signatures and clinical characteristics was constructed to predict the 1-, 3-, and 5-year survival rates of patients.

### Prediction of immunotherapy response

The gene mutation analysis was carried out to determine the quantity and quality of gene mutations among the different groups. The tumor mutational burden (TMB) and tumor immune dysfunction and exclusion (TIDE) were applied to predict differences in immunotherapeutic responses between the different groups. The Kaplan–Meier survival analysis was performed to examine survival differences among different groups.

### Characterization of potential compounds

To explore potential compounds for HNSCC treatment in the clinic, we calculate the half inhibitory concentration (IC50) of compounds and make comparisons in the IC50 between different groups.

### Cell culture

The TU686 and SCC25 cell lines were purchased from The iCell Bioscience Inc,Shanghai (iCell-h300 and iCell-h361, Shanghai, China). The WSU-HN30 cell lines were purchased from Shanghai Zephyr Biotechnology Co. (ZYH60432,Shanghai, China).

The cell TU686 lines were cultured in RPMI 1640 (Gibco, New York, NY, United States) with 10% fetal bovine serum (FBS, Gibco), while SCC25 and WSU-HN30 cell lines were cultured in DMEM (Gibco, New York, NY, United States) supplemented with 10% FBS (Gibco) serum. These cell lines were cultured in a medium supplemented with penicillin–streptomycin (each at 100 Units/ml, MedChemExpress, Shanghai, China) at 37 °C in an incubator with humidifified atmosphere and 5% CO2.

### Western blotting

TU686/SCC25/WSU-HN30 cells were treated with RIPA lysate buffer containing a protease inhibitor (Solarbio, Beijing, China) and a phosphatase inhibitor (Solarbio). Concentrations of proteins within supernatants were determined using a BCA kit (Dowobio, Shanghai, China). Proteins (20 μl per lane) and Marker (5 μl per lane) ,DW1106 (Dowobio, Shanghai, China) were loaded into wells of SDS-PAGE gels followed by electrophoresis to separate proteins by molecular weight (8–10% SDS-PAGE separation gel and a 5% SDS-PAGE concentrated gel). Next, proteins were transferred to polyvinylidene flfluoride membranes (PVDF, Solarbio) using a wet transfer method. PVDF membranes bound to proteins were blocked by immersion in 5% (w/v) bovine serum albumin (Solarbio), then membranes were probed with antibodies anti-Amphigireegulin, ER1903-67; anti-Urokinase Recombinant, ET1703-26, anti-Amyloid Beta A4 Precursor(APP), 1007–5; anti-Caveolin-2, ET1607-15 (HUABIO, Hangzhou, China) as dilutions of 1:1500, GAPDH, ab181602(Dowobio, Shanghai, China), as dilutions of 1:8000,followed by incubation at 4° C overnight. Next, membranes were incubated with secondary antibody (HRP) at room temperature for 1 h. An enhanced ECL,BL523B(Biosharp, AnHui,China) reagent is added to the film, developed after exposure with blue film, fixed, and scanned by a scanner for analysis.

### Quantitative RT-PCR analysis

Total RNA of hippocampus was extracted using Trizol^®^ reagent (Ambion, Waltham, MA, United States), then the quality and concentration of total RNA were determined by 1% agarose gel electrophoresis and Q3000 micro-volume spectrophotometer (Quawell Technology, San Jose, CA, United States). The RevertAid First Strand cDNA Synthesis Kit (Vazyme, Nanjing, China) was used to transfer total RNA into cDNA. Table 1 showed the sequences for primers used in our study which were designed based on published mRNA sequences in NCBI and synthesized by Sangon Biotech Co., Ltd, Shanghai, China. The qRT-PCR reaction system was prepared by SYBR^®^ Green PCR Master Mix (Applied Biosystems) in a final volume of 25 μL, then performed on Multicolor Real-time PCR Detection System (Roche, Basel, Switzerland) with the following thermal cycling conditions: preincubation at 94 °C for 3 min, followed by 40 cycles of denaturation at 94 °C for 30 s, annealing at 55 °C for 30 s, and extending at 72 °C for 30 s. The signals were normalized to GAPDH and the relative expression of mRNA in each sample was calculated by 2^−ΔΔCt^ method.

**Table 1:** The primer sequences used for PCR amplification.

Gene	–	Sequences
PLAU	Froward	GCTATCGGACTGGCTTGAAGA
	Reverse	AGGTAACGGCTTCGGGAATAG
APP	Froward	AGCACACCCTAAAGCATTTCGAG
	Reverse	CGCTCATAAATCACACGGAGGT
AREG	Froward	CATAGCCATAAATGATGAGTCG
	Reverse	GCACCTTTATATACAGAAATAGCA
CAV2	Froward	CCTGCCTAATGGTTCTGCCTTC
	Reverse	GTCCTACGCTCGTACACAATGG

### Ethical approval and consent to participate

All data in this study were collected from public databases: TCGA and GEO. This article does not contain any studies with patients or animals performed by any of the authors.

## Supplementary Information

Supplementary Figure 1.

Supplementary Figure 2.

Supplementary Figure 3.

Supplementary Figure 4.

Supplementary Information 5.

Supplementary Tables.

## Data Availability

The original contributions presented in the study are publicly available. This data can be found here: https://portal.gdc.cancer.gov (TCGA); https://www.ncbi.nlm.nih.gov (GEO).
