# Supplementary material for: Identification of molecular classification and gene signature for predicting prognosis and immunotherapy response in HNSCC using cell differentiation trajectories
Source: Sci Rep. 2022 Nov 27;12:20404. doi: 10.1038/s41598-022-24533-7 (PMC9701758; doi:10.1038/s41598-022-24533-7)
Supplement: Supplementary file 5 — Supplementary Information 5. [file 41598_2022_24533_MOESM5_ESM.doc]

**Supplementary Information**

**Supplementary Table S1.** The differentiation-related genes.

**Supplementary Table S2.** The details of GO and KEGG enrichment analysis for subset I.

**Supplementary Table S3.** The details of GO and KEGG enrichment analysis for subset II.

**Supplementary Table S4.** The details of GO and KEGG enrichment analysis for subset III.

**Supplementary Figure S1.** Gene Ontology and Kyoto Encyclopedia of Genes and Genomes enrichment analysis. (A-B) GO and KEGG enrichment analysis for subset I genes. (C-D) GO and KEGG enrichment analysis for subset II genes. (E-F) GO and KEGG enrichment analysis for subset III genes. BP: Biological Process; CC: Cellular Components; MF: Molecular Function.

**SupplementaryFigure S2.** (A-G ) Consensus matrices of the 11 IRGs for k = 3-9.

**SupplementaryFigure S3.** Identification of common anti-tumor drugs for the model. P < 0.05.

**SupplementaryFigure S4.** The protein expression level of PLAU, APP, AREG and CAV2 were upregulated in cancer cells compared to normal cells.
